# Cyclical Hemoptysis and Pelvic Pain in a Young Female: A Sign of Thoracic Endometriosis Syndrome

**DOI:** 10.7759/cureus.11078

**Published:** 2020-10-21

**Authors:** Areeg Bala, Raghda A Salim, Smit Deliwala, Michele Obeid, Ghassan Bachuwa

**Affiliations:** 1 Internal Medicine, Michigan State University at Hurley Medical Center, Flint, USA; 2 Internal Medicine, University of Khartoum, Khartoum, SDN

**Keywords:** catamenial pneumothorax, catamenial hemothorax, catamenial hemoptysis, video-assisted-thoracoscopy, infertility, dysmenorrhea, thoracic endometriosis syndrome, endometriosis

## Abstract

Distant autoimplantation of endometrial implants presents with signs and symptoms specific to the affected site. The constellation of cyclical hemoptysis, pleuritic chest pain, dyspnea, or cough in the right gynaecologic setting should raise concern for thoracic endometriosis syndrome (TES). Although extra-pelvic implications of endometriosis are well known, TES is exceedingly rare. We present an unusual case of aggressive TES that re-emerged after a period of latency despite suppressive therapy, making the case for future studies to establish surveillance schedules and advanced therapies. As these implants become sizable, they require a combination of medical and surgical therapies often with psychological support. This case illustrates the importance of prompt diagnosis and a multidisciplinary approach to TES.

## Introduction

Endometriosis is a condition where uterine tissue is found in sites outside its cavity, most commonly ovaries, uterosacral ligaments, or the peritoneum. Its incidence ranges between 5%-15% in reproductive females, peaking at 30-34 years [1,2]. As they grow and become clinically and radiographically evident, its signs and symptoms remain vague often masked by menstruation and its syndromes [3]. If unnoticed, they can cause significant morbidity and eventual infertility. When extra-pelvic organs are implicated, patients present with gastrointestinal or genitourinary symptoms from deep seeding of implants into the peritoneum and its surrounding structures [1]. Amongst distant sites, autoimplantation into the thorax is exceedingly rare, often a consequence of long-standing endometriosis, while controlling exsanguination remains a priority on admission. Its recurrence rate is unknown with an inverse impact on mental and physical wellbeing in young patients. These aspects place importance on early detection and control. Once thoracic implants attain a significant size, its clinical manifestations are termed thoracic endometriosis syndrome (TES) [4]. Literature review revealed an inconsistent universal definition and a paucity of recommendations and prospective studies making clinical decision making arbitrary and placing importance on isolated reports to guide future cases. 

## Case presentation

**Figure 1:**
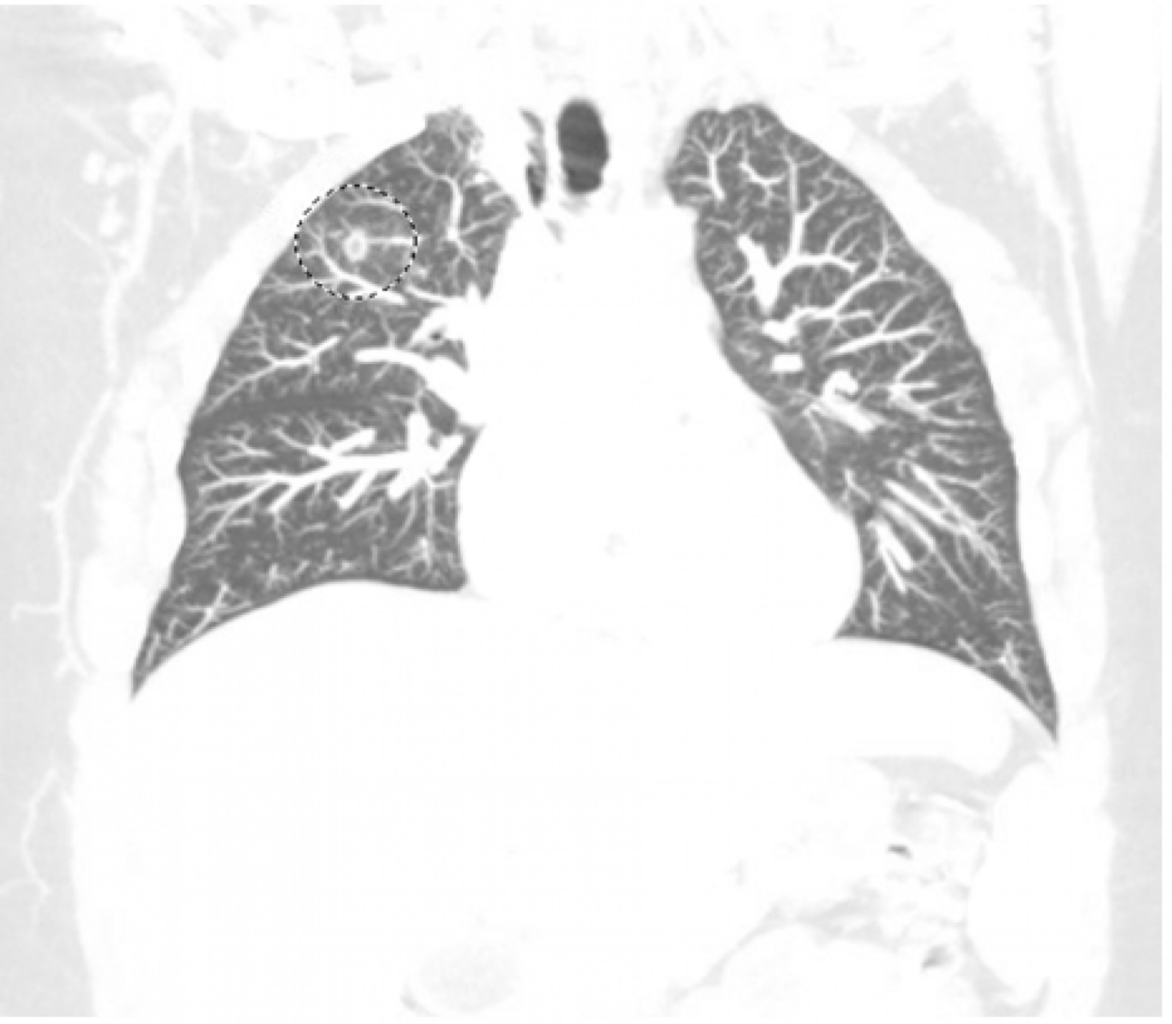
Chest computed tomography (CT) in a coronal view revealing a small focus of ground-glass opacity with a sub-centimeter parenchymal cyst within the right upper lobe (encircled).

**Figure 2:**
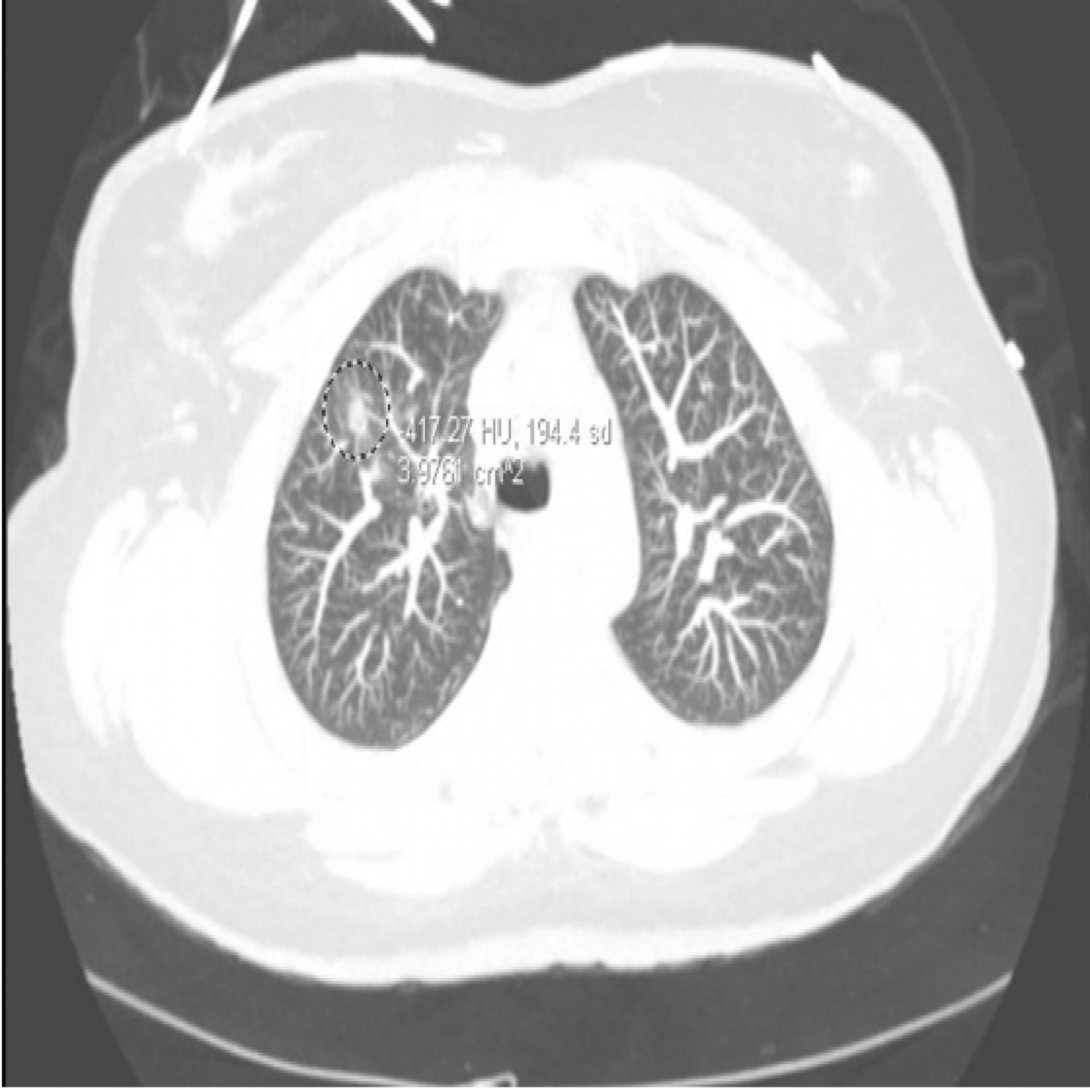
Computed tomography (CT) of the chest in axial view revealing the small focus of ground-glass opacity and its subcentimeter parenchymal cysts within the right upper lobe (encircled).

A 28-year-old female presented to our hospital with severe hemoptysis and stable vital signs evolving over the preceding months. Her symptoms began a few months prior and recurred every month, coinciding with her menstrual cycle. She was reluctant to receive care due to the ongoing coronavirus disease 2019 (COVID-19) epidemic and was largely fearful of becoming sick. She had a complicated history comprising endometriosis requiring multiple laparoscopies, Ehler-Danlos syndrome, seizures, and venous thromboembolism. This was not her first presentation for hemoptysis. However, this was the first presentation in many years after being diagnosed with biopsy-proven endometriosis in the left lower lung by video-assisted thoracotomy surgery (VATS) in her early 20s. However, the foci were deemed too small to resect. At that time, she had presented with similar symptoms and had a prolonged admission that eventually uncovered the cause. She eventually moved states and has been maintained on leuprolide, combination estrogen-progesterone contraceptive pills, enoxaparin, levetiracetam, and folic acid ever since. On exam, she was ill-appearing without any obvious findings apart from blood-stained chuck bed pads. She added that her symptoms have heightened in intensity in the past month since she could not receive her injections due to shortages. A chest tomography with angiography ruled out pulmonary embolism. Still, it revealed a small focus of ground-glass opacity with a sub-centimeter parenchymal cyst within the right upper lobe (Figures 1-2). Laboratory workup revealed anemia with low hemoglobin that did not require transfusion.

**Figure 3:**
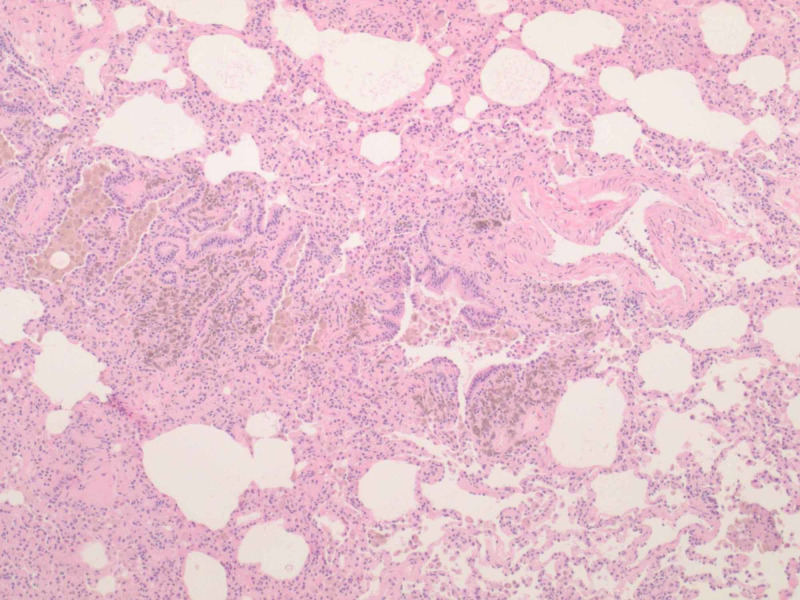
A hematoxylin and eosin (H&E) stain at 40x magnification, revealing patchy pulmonary hemosiderosis and irregular vasculature.

**Figure 4:**
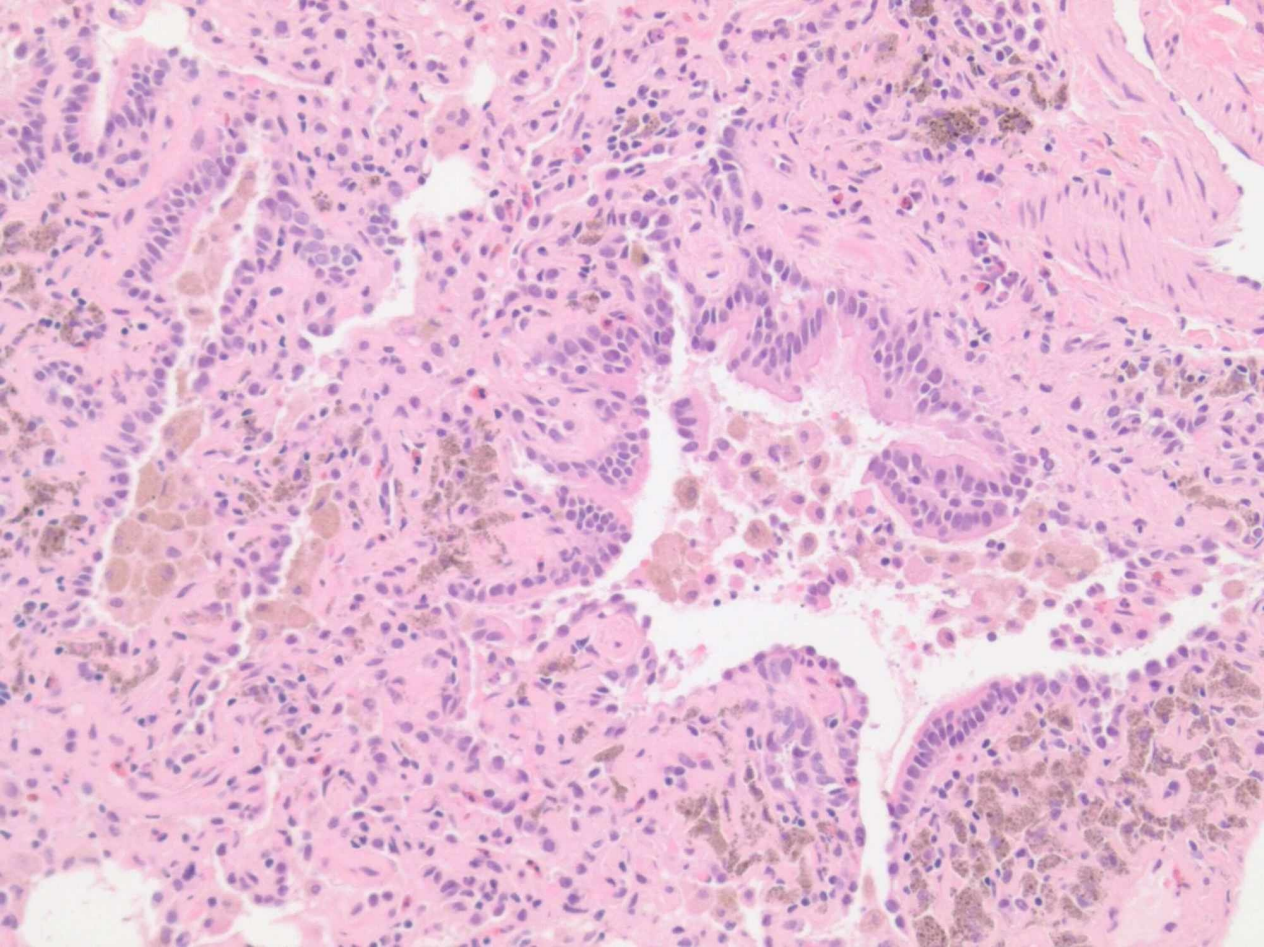
A hematoxylin and eosin (H&E) stain at 100x magnification, revealing patchy pulmonary hemosiderosis and irregular vasculature.

She was transferred to the intensive care unit (ICU) for close monitoring, as her symptoms were still active. Her home medications were restarted, and cardiothoracic surgery was consulted. She was risk-stratified and prepared for surgery, where she underwent wedge excision of the right lobe with muscle-sparing thoracotomy. Biopsy revealed abnormally enlarged vessels with thrombi and surrounding pulmonary hemorrhage and hemosiderosis (Figures 3-4). The decision to forgo lobectomy was made. She had an uneventful postoperative course and was eventually discharged home with appropriate vaccinations. At serial follow-ups, she endorsed that her hemoptysis had significantly improved, although she continued to experience residual post-tussive expectorates of blood during menses.

## Discussion

Endometriosis is a clinical gynecological disorder characterized by functional endometrial tissue in different locations outside the uterine cavity. Its spectrum is broad and requires early suspicion and control. Pain and menstrual bleeding are the main presentations preceded by pelvic pain, while the pain location depends on the site in which ectopic endometrial tissue is present [5]. Accordingly, patients can present with a wide range of presentations, including dysmenorrhea, dyspareunia, dyschezia, and dysuria. Unfortunately, symptoms are not always evident, often uncovered during infertility workups [3]. TES is a rare variant of endometriosis manifesting with chronic chest pain and hemoptysis synchronized to the menstrual cycle, and hence displaying properties of estrogen dependence [4]. TES has four distinct variants that have demonstrated a temporal relationship to menses: catamenial hemothorax, catamenial pneumothorax, catamenial hemoptysis, and pulmonary nodules [5]. Despite a paucity of cases, TES has been known for over a century. Despite advancements in suppressive therapy, its etiology and pathogenesis is complex and poorly understood, with multiple stakeholders contributing to distant implantation. However, several established theories are suggesting how this phenomenon occurs: 1) Retrograde menstruation most commonly affecting the right hemidiaphragm and grossly visualized as fenestrations within the diagram from retrograde movement through the fallopian tubes, 2) Coelomic metaplasia of mesothelial cells under the influence of estrogen, and 3) Benign metastasis through vascular or lymphatic channels that often implant upon bronchopulmonary structures [4]. TES shows a predominance to the right thorax in over 95% of cases due to a combination of anatomic and physiological factors directing fluid and endometrial tissue along the right paracolic gutter into the right subphrenic space [6]. 

The diagnosis of thoracic endometriosis involves the constellation of cyclical signs and supporting image findings [7]. Physical examination may support the diagnosis of pleural effusion and/or pneumothorax, although computed tomography can help exclude other etiologies. Biopsy with histopathologic findings clinches the diagnosis, and an exploratory thoracotomy is usually performed to obtain tissue biopsy [1]. Bronchoscopy can help in localizing the source of bleeding [8]. Concurrent pelvic ultrasound to look for coinciding intra-pelvic endometriosis should be completed as well [8,9]. The basis of TES management is a combination of medical and surgical treatment. Hormonal therapy, including gonadotropin-releasing hormone analogs and danazol, can be used. Despite the proven effectiveness, it is a less favorable approach due to drug cost, side effects, and high rate of recurrence observed in 50% of patients receiving hormonal therapy alone [8-10]. VATS serves multiple purposes with the ability to resect the thoracic implants, obliterate diaphragmatic fenestrations, and eliminate pleural space vacuum by chemical pleurodesis. Refractory cases require more advanced therapies such as lung-sparing segment or wedge lobectomy, similar to the patient presented above.

## Conclusions

TES is an exceedingly rare extra-pelvic variant of endometriosis. The paucity of cases and cohort studies make management arbitrary, placing emphasis on isolated reports. Our patient presented with recurrence of her previously diagnosed TES after a period of latency despite suppressive therapy. Hemoptysis, hemothorax, pneumothorax, and pulmonary nodules are the cardinal thoracic presentations. Hemoptysis is always an emergency for the risk of asphyxia. Management options include hormone suppressive therapy or surgical approaches. Future studies looking into long term outcomes, surveillance schedules, and recurrence will help fill knowledge gaps. Patients must be approached on a case-by-case basis to plan an optimal strategy and maintain fertility status.

## References

[1] Huang H, Li C, Zarogoulidis P (2013). Endometriosis of the lung: report of a case and literature review. Eur J Med Res.

[2] Alwadhi S, Kohli S, Chaudhary B, Gehlot K (2016). Thoracic endometriosis-a rare cause of haemoptysis. J Clin Diagn Res.

[3] Parasar P, Ozcan P, Terry KL (2017). Endometriosis: epidemiology, diagnosis and clinical management. Curr Obstet Gynecol Rep.

[4] Kim M, Hwang H, Namkung J (2018). The estimated prevalence and incidence of endometriosis with administrative data in Korean women: a national population based study. Fertil Steril.

[5] Karpel JP, Appel D, Merav A (1985). Pulmonary endometriosis. Lung.

[6] Azizad-Pinto P, Clarke D (2014). Thoracic endometriosis syndrome: case report and review of the literature. Perm J.

[7] Chatra PS (2012). Thoracic endometriosis: a case report. J Radiol Case Rep.

[8] Kim JH, Park SY (2020). Recurrent hemoptysis in a 26-year-old woman with a ground-glass opacity lesion of the lung. Yeungnam Univ J Med.

[9] Adesanya OA, Kolawole OE (2020). Thoracic endometriosis syndrome: Cutting the gordian knot - a case report and review of the literature. Int J Surg Case Rep.

[10] Nezhat C, Lindheim SR, Backhus L, Vu M, Vang N, Nezhat A, Nezhat C (2019). Thoracic endometriosis syndrome: a review of diagnosis and management. JSLS.

